# Dapagliflozin ameliorates motor deficits in a Parkinson’s disease model induced by 6-OHDA: An integrative *in vivo* and *in silico* approach from α-synuclein/A2AAR/TH/TNF-α to APAF-1/caspase-3 modulation

**DOI:** 10.1007/s10787-026-02241-2

**Published:** 2026-04-18

**Authors:** Selma Sezen, Feyza Burul, Ufuk Okkay, Mehmet Karadayı, Mustafa Ozkaraca, Cemil Bayram, Yusuf Gülşahin, Irmak Ferah Okkay, Medine Gulluce

**Affiliations:** 1 Department of Medical Pharmacology, Faculty of Medicine, Agri Ibrahim Cecen University, Agri, Turkey; 2 Department of Medical Pharmacology, Faculty of Medicine, Ataturk University, Erzurum, Turkey; 3 Department of Biology, Faculty of Science, Ataturk University, Erzurum, Turkey; 4 Department of Pathology, Faculty of Veterinary, Sivas Cumhuriyet University, Sivas, Turkey; 5 Department of Pharmacology and Toxicology, Faculty of Veterinary, Ataturk University, Erzurum, Turkey

**Keywords:** 6-Hydroxydopamine, Dapagliflozin, Molecular docking, Neuroinflammation, Neuroprotective, Parkinson’s disease

## Abstract

Parkinson’s disease (PD), the second most common neurodegenerative disorder, is characterized by α-synuclein aggregation and loss of dopaminergic neurons, and current treatments are symptomatic. Multiple intricate mechanisms contribute to the pathogenesis, and the effectiveness of single-target approaches is therefore limited. Current approaches highlight therapeutic candidates capable of simultaneously modulating multiple pathways. Recent clinical and experimental studies of sodium-glucose cotransporter inhibitors (SGLT2is), approved for the treatment of type 2 diabetes mellitus, have indicated their pleiotropic and neuroprotective potential. SGLT2i dapagliflozin has several features, including low molecular weight, blood-brain barrier permeability, and tolerability. This study investigated the effects of dapagliflozin on pathways implicated in the pathogenesis of PD in a 6-hydroxydopamine-induced experimental PD model in female Sprague-Dawley rats using *in silico*, histopathological, immunohistochemical, and biochemical methods. Dapagliflozin was administered by oral gavage at four different doses (2.5 mg/kg, 5 mg/kg, 7.5 mg/kg, and 10 mg/kg) for 14 days. Motor deficits were evaluated by means of behavioral tests, and dapagliflozin was observed to alleviate motor dysfunction. Tyrosine hydroxylase expression increased in brain tissues, whereas A2AAR, TNF-α, and APAF-1 levels, as well as α-synuclein and caspase-3 expression, decreased. In molecular docking analyses, dapagliflozin showed notable binding affinity to PD-associated human target receptors. Our results suggest that dapagliflozin may exert potential neuroprotective effects via modulation of inflammatory, oxidative stress-related, and apoptosis-associated pathways and may represent a promising repurposing candidate for PD.

**Graphical abstract:**

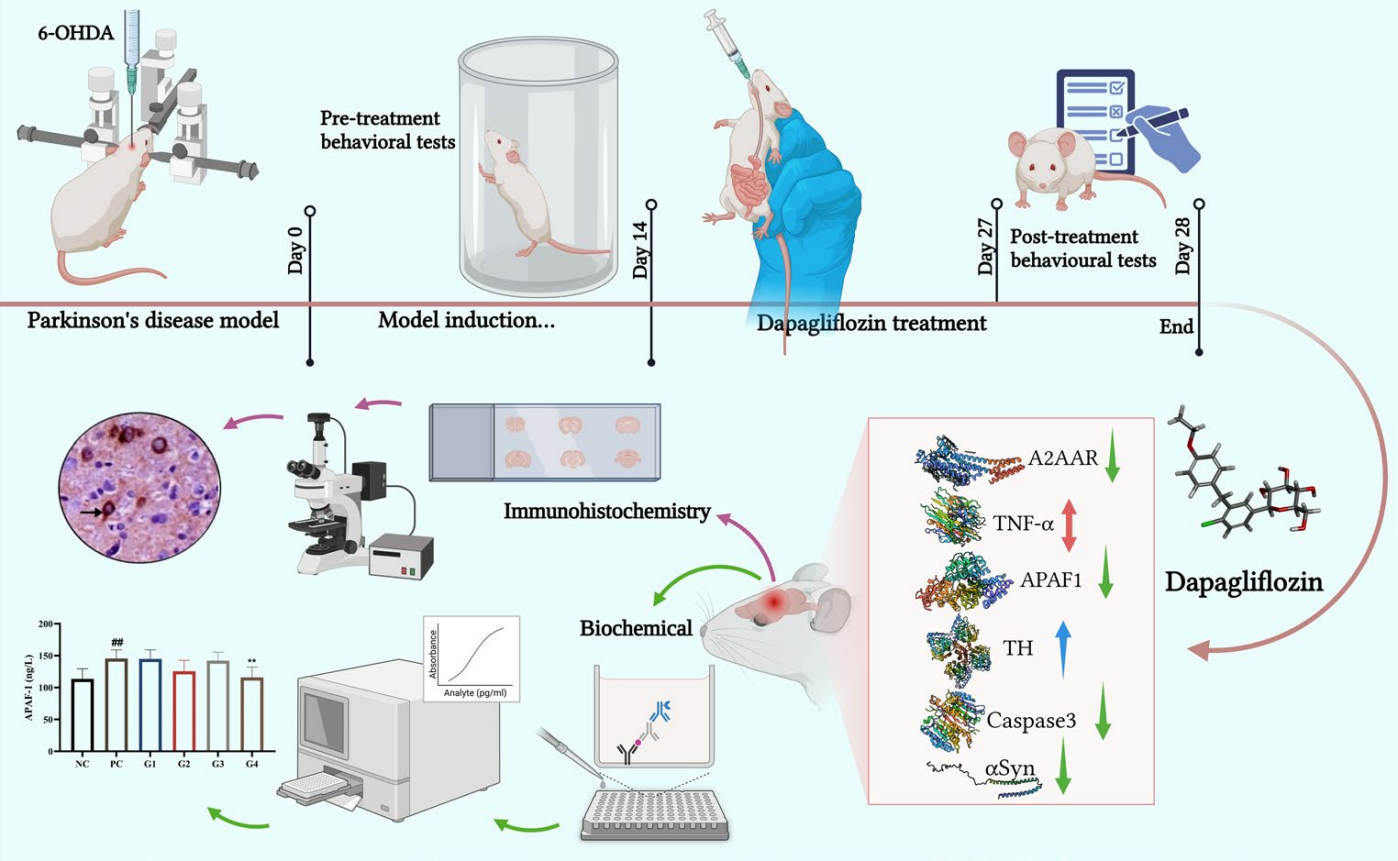

## Introduction

Parkinson’s disease (PD), characterized by degeneration of dopaminergic neurons in the substantia nigra (SN) and affecting the extrapyramidal motor system, is the second most common neurodegenerative disorder (Aludin and Schmill 2021; Zhu et al. 2022; Soni et al. 2024). Motor deficits (akinesia, rigidity, postural instability, and tremor) and non-motor deficits (hallucinations, paresthesia, and sleep and cognitive disturbances), manifested when dopaminergic neuron loss reaches approximately 60%, adversely affect patients’ quality of life (Aryal et al. 2020; Balestrino and Schapira 2020; Tolosa et al. 2021). In its 2023 report, the World Health Organization (WHO) indicated that the prevalence of PD had doubled over the previous 25 years, while published estimates suggested that this number may reach 12 million by 2040 as the population ages (Dorsey et al. 2018; WHO, 2023). Although the disease is progressive, the limited efficacy of current treatments and their provision of only symptomatic relief make it highly important to elucidate the mechanisms underlying PD and to develop novel therapeutic approaches (Stoker et al. 2018; O’Mahony et al., 2025).

The protein α-synuclein, which is well defined in the pathophysiology of PD, accumulates in the central nervous system, where it forms aggregates. These α-synuclein aggregates, which activate microglia, have been shown to exacerbate neuroinflammation through various pro-inflammatory cytokines, particularly tumor necrosis factor (TNF)-α, and to lead to striatal neuronal degeneration (Calabresi et al. 2023; Hassanzadeh et al. 2024). In addition, overexpression of α-synuclein has been shown to cause a decrease in tyrosine hydroxylase (TH) activity in the striatal region, thereby directly damaging the dopaminergic system (Calabresi et al. 2023; Manca et al. 2025). Post-mortem brain tissue specimens from patients with PD have also been reported to exhibit a marked decrease in TH activity in the nigrostriatal system (Rausch et al. 2022). Impairment of TH activity leads to the formation of reactive metabolites, which conjugate with α-synuclein and promote aggregate formation. This process triggers neurotoxicity that results in cognitive dysfunction, and also significantly disrupts the dopaminergic system balance responsible for the deterioration of patients’ motor functions (Yi et al. 2024).

Two pathways responsible for dopaminergic transmission are known to play critical roles in the regulation of motor functions. Dopamine D1 receptors play a central role in the direct pathway, which facilitates the initiation of voluntary movements. However, initiating voluntary movement alone is not sufficient for motor control, and suppression of unwanted movements is also required. In the indirect pathway, which mediates this function, dopamine D2 and adenosine A2A receptors (A2AAR) are co-expressed, and A2AAR is particularly prominent in regulating signaling associated with involuntary movements. In the indirect pathway, D2 receptor signaling suppresses activation, while A2AAR activates the pathway. D2 and A2AAR thus act in opposition to one another. In the case of PD, despite reduced dopaminergic input, overexpression of A2AAR leads to abnormal overactivation of the indirect pathway and attenuation of the movement-facilitating effect of the direct pathway, resulting in bradykinesia and other motor impairments (Mori et al. 2022; Pinna et al. 2018). Increased A2AAR signaling has also been reported to exacerbate glutamatergic excitotoxicity and the microglial inflammatory response. A recent study reported that compounds synthesized as A2AAR antagonists improved motor function in rats in a 6-hydroxydopamine (6-OHDA)-induced experimental PD model (Spinaci et al. 2025). Approaches suggesting that A2AAR antagonists may enhance dopaminergic signaling and alleviate neuroinflammation are among the therapeutic strategies employed in PD (Kanda and Jenner 2020; Jost and Tönges 2022).

Increased mitochondrial dysfunction in PD, resulting from inflammatory and receptor-mediated signaling disturbances, also triggers the activation of apoptotic pathways involved in cell death. In the intrinsic mitochondrial apoptotic pathway, apoptotic protease-activating factor 1 (APAF-1) is normally an inactive protein. However, following binding of cytochrome c released in the presence of oxidative stress and mitochondrial dysfunction, it becomes activated and forms multiprotein complexes. These structures, known as apoptosomes, activate caspases involved in neurodevelopmental cell death. Caspase-3, which causes irreversible apoptotic damage in dopaminergic neurons, has been described as the caspase most prominently upregulated at the transcriptional level in response to neuronal injury. Moreover, increased caspase-3 activity has been demonstrated in post-mortem human brain tissues (Erekat 2018; Wang et al. 2018; Hollville et al. 2019; García-Revilla et al. 2024). A study involving an experimental PD model noted that treatment reduced (1-methyl-4-phenyl-1,2,3,6-tetrahydropyridine (MPTP)-induced increases in APAF-1 and caspase-3 expression. The observed neuroprotective effect was associated with attenuation of mitochondrial dysfunction (Wang et al. 2018). Inhibition of APAF-1 and caspase-3, whose expression levels have been shown to rise in association with oxidative stress and neuroinflammation, is considered a potential therapeutic target for PD (Chang et al. 2023; Pedrão et al. 2024).

L-3,4-dihydroxyphenylalanine (L-DOPA), dopamine agonists, monoamine oxidase B (MAO-B) inhibitors, and catechol-O-methyltransferase (COMT) inhibitors, aimed at enhancing dopaminergic activity, are currently used in the treatment of PD, together with anticholinergic drugs such as trihexyphenidyl and biperiden, intended to rebalance the disrupted cholinergic system. However, in addition to being symptomatic, these therapies can also lead to problems such as motor fluctuations, dyskinesias, and various non-motor adverse effects, thereby limiting their clinical use and long-term efficacy (Freitas et al. 2017; Hamed and Hadad 2024). In addition, the complex pathogenesis of PD, which involves protein aggregation, inflammation, oxidative stress, and dysregulation of apoptotic processes, has made it difficult to identify a single therapeutic target (More and Choi 2016). In that context, research is taking place into the development of new therapeutic strategies capable of modulating multiple pathological pathways and targeting neuroinflammation, oxidative stress, and apoptotic processes, which play critical roles in PD.

Recent studies provide substantial evidence that a new generation of antidiabetic sodium-glucose cotransporter inhibitors (SGLTis), which are frequently used in the treatment of type 2 diabetes and exhibit pleiotropic effects, may alleviate neurodegeneration. Sodium-glucose cotransporters (SGLTs) are membrane proteins that transport various hexoses, primarily glucose, together with sodium ions into cells. Hyperactivation of these transporters has been associated with degeneration exceeding chronic hyperglycemia, through effects on oxidative stress, inflammatory responses, and vascular functions. In particular, medium- and long-acting SGLTis have been reported to reduce oxidative stress markers in addition to improving glycemic control, to restore mitochondrial function, and to exert neuroprotective effects by suppressing signaling pathways involved in neuroinflammatory responses (Lin et al. 2014; Yaribeygi et al. 2019; Hsieh and Sung 2022; Mohammed et al. 2024; Sezen and Burul 2026). Dapagliflozin, which exhibits high selectivity for SGLT2, is a long-acting SGLT2i that reduces HbA1c more markedly than other oral antidiabetic agents (Rizzo et al. 2022). Moreover, owing to its low molecular weight and lipophilic structure, dapagliflozin has been reported to cross the blood–brain barrier (BBB), an important obstacle to the effective treatment of neurodegenerative diseases, and to exert neuroprotective effects in neurodegenerative disorders such as Alzheimer’s disease (AD) and Huntington’s disease (El-Sahar et al. 2020; Nguyen et al. 2020; Wu et al. 2023; Sezen 2025). In a large-scale cohort study conducted between 2016 and 2020, SGLT2i use in patients with type 2 diabetes was associated with a reduced risk of dementia; dapagliflozin was identified as the agent within this class associated with the lowest dementia risk (Wu et al. 2023). To the best of our knowledge, the effects of dapagliflozin on PD have not been systematically evaluated in a comprehensive *in vivo* model. Although the potential beneficial effects of dapagliflozin on neurodegeneration in dementia with Lewy bodies and PD are currently being investigated in an ongoing Phase 4 clinical trial, no registered clinical outcomes from that trial have to date been reported in ClinicalTrials.gov or related databases (NCT06263673). While a Phase 4 study is being conducted by other investigators, the present study constitutes the first report to systematically evaluate the neuroprotective effects of dapagliflozin on neuroinflammation, oxidative stress, and apoptotic pathways in a 6-OHDA-induced experimental PD model.

## Materials and methods

### In silico analyses

#### Protein-protein interaction network construction and functional enrichment analysis

Previous experimental and clinical studies have demonstrated that multiple complex mechanisms, including protein aggregation, neuroinflammation, dopaminergic dysfunction, and apoptotic responses, contribute to the pathogenesis of PD. Accordingly, critical PD-associated target proteins, α-synuclein, A2AAR, TH, TNF-α, APAF-1, and caspase-3, were identified through a literature review (Mori et al. 2022; Wang et al. 2018; Yi et al. 2024; Pan et al. 2025). In order to evaluate the positions of these targets within the known interaction network related to human PD and their shared network connections, a protein-protein interaction (PPI) network was constructed using the STRING database (https://string-db.org). For network construction, the target proteins were entered into STRING, “*Homo sapiens*” was selected as the organism, the network parameters being defined as network type: “full STRING network”, meaning of network edges: “evidence”, and minimum required interaction score: 0.400 (medium confidence). Network statistics for the PPI network were obtained from the STRING database (Sezen and Burul 2026).

An enrichment analysis including Gene Ontology (GO, Biological Process) terms was performed using the functional enrichment module of the STRING database to identify the major signaling pathways associated with the target proteins in the PPI network. GO terms with > 80% similarity in gene compositions were grouped under the same functional cluster. Statistical significance was assessed using an FDR-adjusted threshold of *p* < 0.05 (Mugundan et al. 2025; Sezen and Burul 2026).

#### Molecular docking analysis

Following the identification of human-derived target proteins and their interactions through STRING analyses, the potential binding interactions of dapagliflozin with these proteins were evaluated using molecular docking methods. α-synuclein (1XQ8), A2AAR (4EIY), TH (2XSN), TNF-α (2AZ5), APAF-1 (1Z6T), and caspase-3 (3DEI) were selected as PD-associated receptors, their molecular structures being obtained from the RCSB Protein Data Bank. The 3D molecular structures of ligands including dapagliflozin (CID: 9887712) and model inhibitors of the PD-associated receptors A2AAR antagonist-1 (CID: 105526064), SPD-304 (CID: 5327044), ZYZ-488 (CID: 138454738), metyrosine (CID: 441350), Z-DEVD-FMK (CID: 16760394), and emrusolmin (CID: 44608289) were downloaded from PubChem, a freely accessible database containing validated information on chemical structures. The receptors and ligands for molecular docking simulations were prepared using Protein Preparation Workflow, LigPrep, and Receptor Grid Generation tools of Schrodinger. Docking studies were performed with Schrodinger Glide and visualized using Schrodinger Maestro version 14.3.129 (Sezen et al. 2025).

#### Animals and study design

Sprague–Dawley rats, which are widely used in experimental studies, are frequently preferred for mimicking human diseases because of their suitability for genetic manipulation as well as their biological similarities and genetic background comparable to those of humans (Khan et al. 2024a). In this study, approval for the experiments was obtained from the Atatürk University Local Ethics Committee for Animal Experiments (HADYEK) (protocol number: 150, 24.06.2024). In accordance with the Animal Research: Reporting of In Vivo Experiments (ARRIVE) guidelines, the animals were acclimatized for one week before the experiments commenced. Efforts were made to reduce animal stress (Percie du Sert et al. 2020).

The sample size was determined by power analysis using WSSPAS: Web-Based Sample Size & Power Analysis Software, and Wang et al.‘s study was taken as a reference (Arslan et al. 2018; Wang et al. 2021). Based on an alpha level of 0.05, a power of 0.80, and an effect size of 0.67, the minimum required sample size was calculated as 6 animals per group, corresponding to a total of 36 animals for six groups. Thirty-six female Sprague–Dawley rats aged 16 weeks and weighing 250–300 g were housed in standard transparent wire-top cages with six rats per cage according to randomized group allocation at 22 ± 3° C under a 12-h dark/12-h light cycle and allowed ad libitum access to standard laboratory chow and tap water.

The animals were randomly assigned to six experimental groups (*n* = 6 per group) using a simple randomization approach before model induction and treatment administration. The study design comprised two control groups and four treatment groups to evaluate the effects of dapagliflozin in an experimental PD model. The PD model was induced with 6-OHDA. The groups were designated as negative control (NC; no PD model induction and no treatment), positive control (PC; PD model induction without treatment), G1 (6-OHDA + dapagliflozin 2.5 mg/kg), G2 (6-OHDA + dapagliflozin 5 mg/kg), G3 (6-OHDA + dapagliflozin 7.5 mg/kg), and G4 (6-OHDA + dapagliflozin 10 mg/kg); the efficacy of dapagliflozin treatment was evaluated relative to the PC group (Abdelhafez et al. 2025).

No animals or data points were excluded from the study or from the statistical analyses. Only the principal investigator and the researcher administering dapagliflozin were aware of the group allocation during treatment administration. Investigators involved in outcome assessment and data analysis were blinded to group allocation. During the postoperative period, the animals were monitored daily for general condition, cage maintenance, and wound site status, and postoperative care was provided throughout the study.

#### Stereotaxic surgery

The rats were anesthetized before surgery with intraperitoneal (i.p.) xylazine (10 mg/kg, Rompun^®^ 2%, Bayer, Germany) and ketamine (60 mg/kg, Keta-Control^®^ 100 mg/mL, Doğa İlaç, Türkiye) (Sezen et al. 2026). In order to establish the PD model, rats in all groups, except for the NC group, received a stereotaxic injection of 32 µg/4 µL 6-OHDA prepared in saline solution containing 0.1% ascorbic acid into the right substantia nigra pars compacta (SNpc) at the following coordinates: anteroposterior (AP) −5.5 mm (from the bregma), lateral (L) 2 mm (from the midsagittal suture), and dorsoventral (DV) 8 mm (from the skull surface). In order to prevent backflow of 6-OHDA, the injection was administered slowly to the target region using a Hamilton syringe at a rate of 1 µL/min. Following completion of all procedures, the surgical site was closed appropriately. The rats were monitored for 14 days after surgery to allow establishment of the PD model (Aydın et al. 2026).

### Behavioral tests

#### Apomorphine-induced rotation test

The apomorphine-induced rotation test was performed by the administration of apomorphine, which elicits contralateral rotational behavior in the direction opposite to the lesioned side in the striatum. The test was conducted at two time points, once 14 days after model establishment to confirm the 6-OHDA-induced PD model, and again following 14 days of oral dapagliflozin treatment to evaluate its therapeutic efficacy. The rats were placed into transparent Plexiglass cylinders and allowed to habituate for 5 min, after which apomorphine (2.5 mg/kg, i.p.) was administered. Their movements were recorded via a video camera for 30 min. Ipsilateral and contralateral rotations were counted from the video recordings, and the number of contralateral rotations was used for the analyses. The results were finally subjected to statistical analysis (Moradganjeh et al. 2013).

#### Open field test

The locomotor activity tests were performed at three different time points (at baseline, 14 days after PD model induction, and following 14 days of oral dapagliflozin treatment to evaluate dapagliflozin efficacy) in order to determine changes in motor function. Plexiglass cages measuring 42 × 42 × 42 cm (May Act 508) were used for the locomotor activity measurements. The experiment was conducted in a quiet environment to prevent the rats’ behavior from being affected by external stimuli. After each measurement, the area was cleaned with 70% ethanol to prevent odor traces from affecting subsequent measurements. Each rat’s stereotypic, vertical, and ambulatory activities, resting times, and distances traveled over 10 min were recorded by the device on a computer. The data obtained were then subjected to statistical analysis (Yörük et al. 2022; Bayraktar et al. 2024).

#### Cylinder test

The cylinder test is a simple and sensitive method for detecting motor impairments in PD models that other behavioral tests may not capture. The test was performed at two different time points, once 14 days after 6-OHDA administration and again on day 14 of dapagliflozin treatment. The rats were placed in a transparent Plexiglass cylinder 20 cm in diameter and 40 cm in height. Each animal was recorded for 30 min using a video camera and was evaluated individually. The rate of right paw use was calculated by counting the number of right and left forelimb contacts made by each rat using the formula [right/ (left+ right)] × 100 (Rattka et al. 2016; Magno et al. 2019).

#### Oral dapagliflozin treatment

The dose range for dapagliflozin treatment was determined by considering doses of other SGLT2is that have exhibited neuroprotective effects in experimental PD models. Fourteen days after surgery, animals in the G1, G2, G3, and G4 groups received dapagliflozin (Forxiga^®^ 10 mg, AstraZeneca) at doses of 2.5 mg/kg, 5 mg/kg, 7.5 mg/kg, and 10 mg/kg, respectively, administered once daily by oral gavage for 14 days. Animals in the PC group received saline at the same volume for the same duration, while no oral treatment was administered to the NC group (Arab et al. 2021; Motawi et al. 2022; Mohammed et al. 2024).

#### Tissue collection and processing

At the end of day 28 following 6-OHDA administration, the rats were sacrificed under deep anesthesia (xylazine 50 mg/kg and ketamine 150 mg/kg, i.p.). Brain tissues were then carefully removed for biochemical, histopathological, and immunohistochemical analyses. Tissues to be used for biochemical analyses were stored at −80° C, while those allocated for histopathological and immunohistochemical examinations were preserved in 10% neutral formaldehyde solution. Rat brain tissues were used in all analyses (Okkay et al. 2021).

### Histopathology

Rat brain tissues fixed in 10% neutral formalin solution were processed through routine alcohol-xylol procedures and embedded in paraffin blocks for histopathological examination. Section  (5 μm) mounted on poly-L-lysine-coated slides were stained with hematoxylin and eosin. Pyknotic and degenerative changes observed in neurons were semi-quantitatively scored as none, mild, moderate, severe, and very severe (Toraman et al. 2023).

### Immunohistochemistry

Briefly, 5 μm tissue sections mounted on poly-L-lysine-coated slides were deparaffinized through xylene and graded alcohol series for immunohistochemical examination. After washing with phosphate-buffered saline (PBS), endogenous peroxidase activity was blocked by incubation in 3% H_2_O_2_ for 10 min. For antigen retrieval, the sections were treated with antigen retrieval solution at 500 W for 2 × 5 min to unmask antigens. Following washing with PBS, the sections were incubated overnight at 4° C with primary antibodies against α-synuclein (Santa Cruz, Cat. No. sc-69977, 1:100 dilution), TH (Affbiotech, Cat. No. AF6113, 1:200 dilution), and caspase-3 (Elabscience, Cat. No. E-AB-30004, 1:200 dilution). The Large Volume Detection System: anti-Polyvalent, HRP (Thermo Fisher, Cat. No. TP-125-HL) was used as the secondary detection system according to the manufacturer’s instructions. 3,3′-diaminobenzidine (DAB) was used as the chromogen at 40 µl/ml concentration. After counterstaining with Mayer’s hematoxylin, the sections were mounted with Entellan and examined under a light microscope. Immunopositivity in six randomly selected fields in the SN was evaluated as absent (−), mild (1–3 cells), moderate (4–5 cells), and severe (> 6 cells) (Khan et al. 2023; Sezen et al. 2026).

### Biochemical analysis

The levels of A2AAR (synonym ADORA2A) (Cat. No. E1844Ra), TNF-α (Cat. No. E0764Ra) and APAF-1 (Cat. No. E1413Ra), associated with neuroinflammation, were measured in rat brain tissue using ELISA kits in accordance with the manufacturer’s protocol (Sezen et al. 2022; Gundogdu et al. 2026).

### Statistical analysis

Data obtained from histopathological and immunohistochemical analyses were analyzed using IBM SPSS Statistics 20.00. Differences among groups were assessed with the nonparametric Kruskal-Wallis test, the groups responsible for the differences being identified using the Mann-Whitney U test. Data from biochemical analyses and behavioral tests were analyzed using GraphPad Prism 9.0. Before performing parametric analyses, normality of data distribution was assessed using the Shapiro-Wilk test. Since all groups passed the normality test (*p* > 0.05), one-way ANOVA followed by appropriate post hoc tests was applied. All results were expressed as mean ± standard deviation (SD), and *p* < 0.05 was considered statistically significant (Sezen et al. 2023).

## Results

### PD-related targets form a highly connected PPI network enriched in dopaminergic and apoptotic pathways

**Fig. 1:**
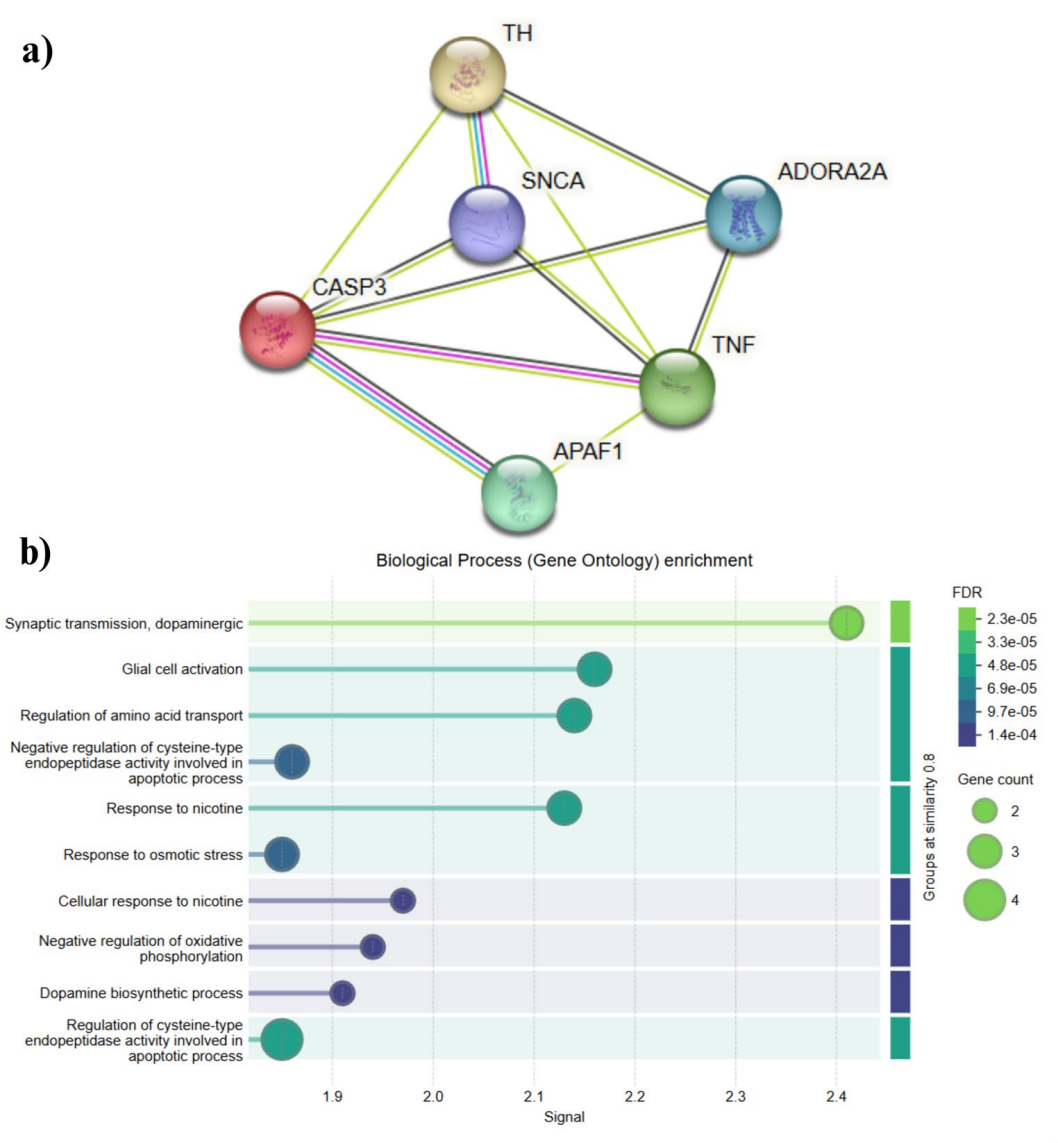
**a** Interaction networks of PD-related proteins (SNCA, ADORA2A (synonym A2AAR), TH, TNF, APAF-1, CASP3) selected as therapeutic targets, constructed using the STRING database. **b** Biological Process (Gene Ontology) enrichment analysis of PD-related proteins (SNCA, ADORA2A (synonym A2AAR), TH, TNF, APAF-1, CASP3) performed using the STRING enrichment interface

Interactions among PD-associated α-synuclein, ADORA2A (synonym A2AAR), TH, TNF-α, APAF-1, and caspase-3 proteins were evaluated using STRING-based network analyses (Fig. 1). Although three edges were expected for the constructed protein network, 11 were in fact observed; the number of nodes was six, the average node degree was 3.67, and the average local clustering coefficient was 0.839. In this protein network, observed to be tightly connected within a functional module, the PPI enrichment *p*-value was recorded as 7.58 × 10^−8^, indicating a statistically significantly dense interaction network. According to the GO enrichment analysis results, at least three proteins were associated with dopaminergic synaptic transmission with a signal greater than 2.4, and the FDR for this association was recorded as 2.3 × 10^−5^. In addition, this network was primarily associated with processes related to glial cell activation, dopamine biosynthesis, and apoptosis (Fig. 1).

### PD-associated α-synuclein, A2AAR, TH, TNF-α, APAF-1, and caspase-3 receptors exhibit high binding affinities with dapagliflozin

**Fig. 2:**
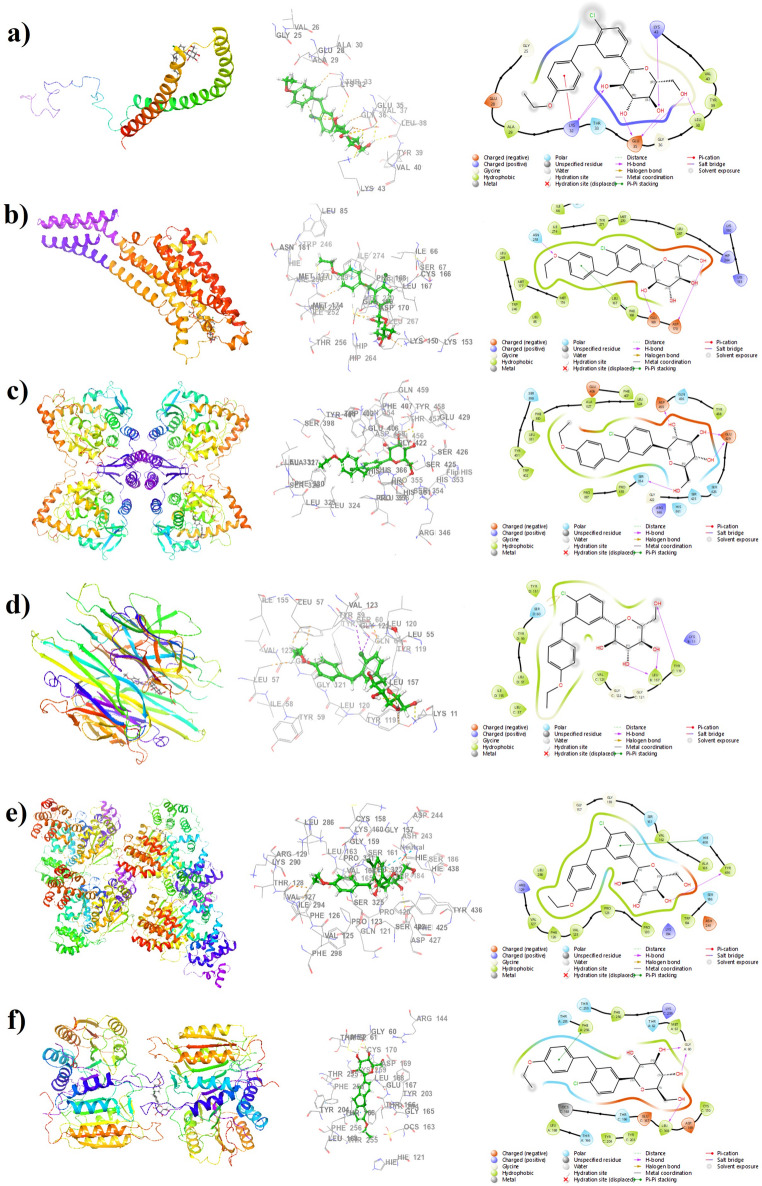
Molecular interactions between dapagliflozin and the PD associated target receptors: **a** α-synuclein and dapagliflozin complex, **b** A2AAR and dapagliflozin complex, **c** TH and dapagliflozin complex, **d** TNF-α and dapagliflozin complex, **e** APAF-1 and dapagliflozin complex, **f** Caspase-3 and dapagliflozin complex

The results of *in silico* molecular docking and simulation studies showed that dapagliflozin has a significant binding affinity to the PD-associated target receptors α-synuclein, A2AAR, TH, TNF-α, APAF-1, and caspase-3. According to the results, dapagliflozin formed three H bonds and a π–π stacking interaction with the crystal structure of A2AAR, two halogen and three H bonds with TNF-α, one H bond and π–π stacking interaction with APAF-1, four H bonds with TH, three H bonds and π–π stacking interaction with caspase-3, and seven H bonds and π-cation interaction with α-synuclein. All molecular docking results for dapagliflozin were comparable to the model inhibitors of PD-associated receptors. The molecular docking scores and detailed comparative interactions are given in Table 1 and visualized in Fig. 2.

**Table 1:** Physico-chemical properties of dapagliflozin docked with PD associated receptors

Receptor	Receptor ID	Ligands (Model Inhibitor vs. Dapagliflozin)	Interacting Residues	Distance (Å)	Interaction	Docking Score (kcal/mol)
α-synuclein	1XQ8	Emrusolmin	LYS97	1.66	H-Bond	−3.726
		Dapagliflozin	LYS32 LYS32 LYS32 GLU35 GLU35 GLU35 LYS43 LEU38	4.32 1.91 2.64 1.78 2.12 2.54 2.28 2.08	π-cation H-Bond H-Bond H-Bond H-Bond H-Bond H-Bond H-Bond	−4.126
A2AAR	4EIY	A2AAR antagonist-1	PHE168 PHE168 TYR271 GLU169 ASN253	3.78 3.91 5.29 2.07 1.83	π-π stacking π-π stacking π-π stacking H-Bond H-Bond	−9.203
		Dapagliflozin	PHE168 ASN253 ASP170 GLU169	3.94 2.00 1.82 2.33	π-π stacking H-Bond H-Bond H-Bond	−7.387
TH	2XSN	Metyrosine	ASP455 GLU406 SER426	2.08 1.89 2.55	H-Bond H-Bond H-Bond	−5.605
		Dapagliflozin	ASP455 GLU429 GLU429 SER354	1.96 1.72 1.85 2.00	H-Bond H-Bond H-Bond H-Bond	−5.476
TNF-α	2AZ5	SPD-304	TYR59	4.33	π-π stacking	−7.625
		Dapagliflozin	SER60 SER60 LEU157 LEU157 TYR119	3.35 3.47 1.75 1.89 2.37	Halogen Bond Halogen Bond H-Bond H-Bond H-Bond	−5.771
APAF-1	1Z6T	ZYZ-488	SER325 ASP392	1.96 1.80	H-Bond H-Bond	−6.144
		Dapagliflozin	HIE438 TYR436	5.06 1.82	π-π stacking H-Bond	−5.802
Caspase-3	3DEI	Z-DEVD-FMK	ARG144 ARG144 LEU168 THR166	3.88 2.11 2.58 2.07	Salt Bridge H-Bond H-Bond H-Bond	−6.241
		Dapagliflozin	PHE256 GLY60 GLY60 LEU168	5.20 1.80 2.27 1.81	π-π stacking H-Bond H-Bond H-Bond	−6.055

### Oral dapagliflozin treatment modulates 6-OHDA-induced motor dysfunction

**Fig. 3:**
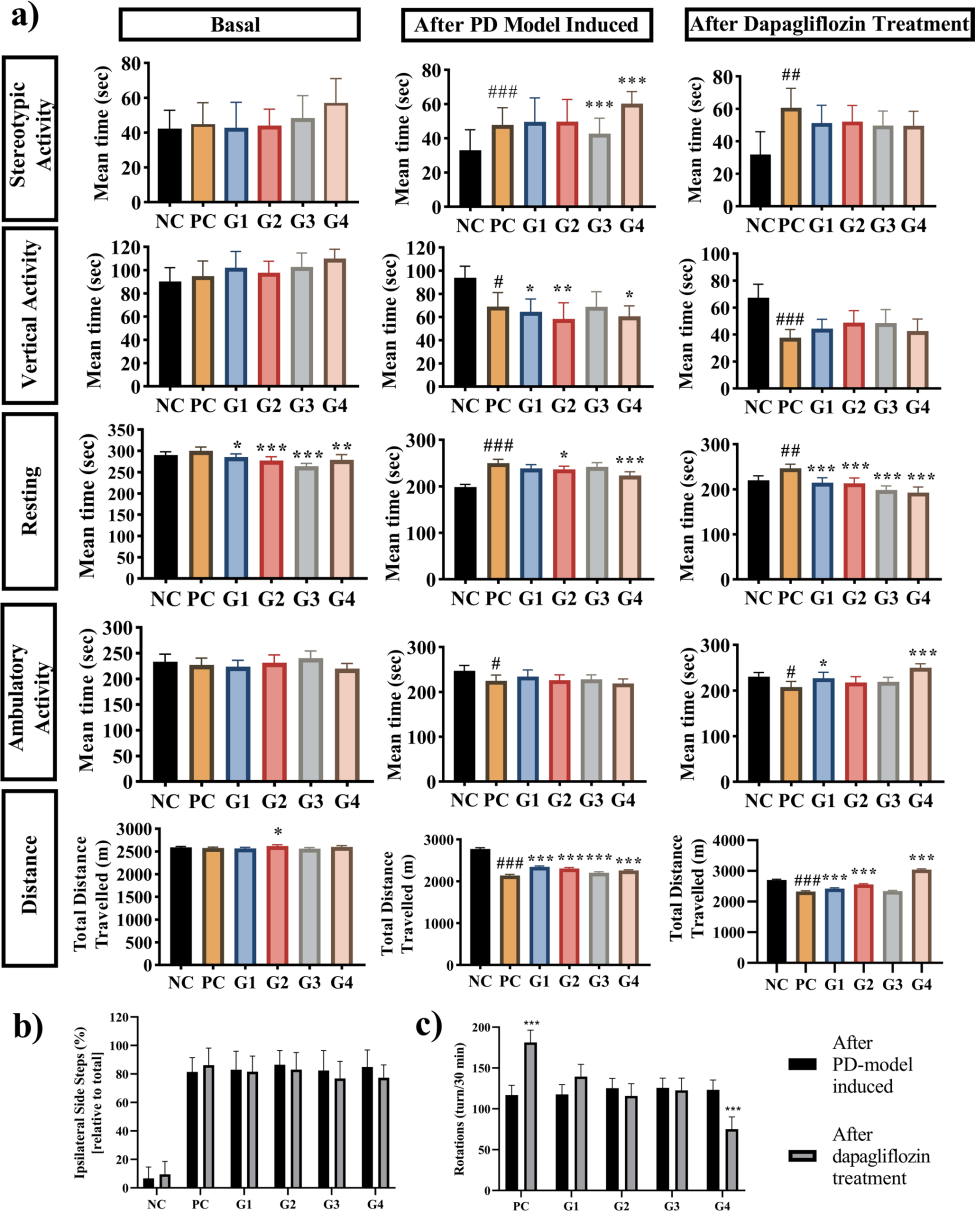
Effect of oral dapagliflozin treatment on motor function in rats: **a** open-field test, parameters assessed were stereotypic activity, vertical activity, resting time, ambulatory activity and distance. Data were recorded at three stages: basal, after PD model induction, and after dapagliflozin treatment (One-way ANOVA with post-hoc Tukey test). **b** cylinder test, parameters assessed were ipsilateral side steps (%). Data were recorded at two stages: After PD model induction, and after dapagliflozin treatment. **c** apomorphine-induced rotation test, parameters assessed were rotations in 30 min. All data are presented as mean ± SD (*n* = 6 animals per group). ^#^*p* < 0.05, ^##^*p* < 0.01, ^###^*p* < 0.001 compared to NC, ^*^p < 0.05, ^**^p < 0.01, ^***^p < 0.001 compared to PC. Negative control (NC), positive control (PC), 6-OHDA + dapagliflozin 2.5 mg/kg (G1), 6-OHDA + dapagliflozin 5 mg/kg (G2), 6-OHDA + dapagliflozin 7.5 mg/kg (G3) and 6-OHDA + dapagliflozin 10 mg/kg (G4)- (Two-way ANOVA with post-hoc Tukey test)

Baseline assessments of the locomotor activity tests showed that stereotypic, vertical, and ambulatory activities, as well as total distance traveled, were similar across all the groups. In post-model measurements, all the groups administered 6-OHDA exhibited decreased vertical activity, ambulatory activity, and total distance traveled values compared with the NC group, with a correlated significant increase in resting time (#*p* < 0.05, ###*p* < 0.001). Stereotypic movement duration increased in all the 6-OHDA-lesioned groups compared with the NC group. However, only the G3 group exhibited a significant reduction relative to the PC group. Following dapagliflozin treatment, resting times were significantly lower in all the treatment groups compared with the PC group, and locomotor performance registered a partial improvement (***p < 0.001). Ambulatory activity in the G2 and G3 groups remained similar to that in the PC group, whereas the G4 group exhibited significant increases in ambulatory activity and total distance traveled, and also the highest motor activity (***p < 0.001). Vertical activity also tended to increase post-treatment in the treatment groups compared with the PC group, although this trend did not reach statistical significance (Fig. 3a).

The cylinder test was performed to confirm the establishment of the model in rats after 6-OHDA administration and to evaluate the effect of dapagliflozin treatment on 6-OHDA-induced forelimb asymmetry. An increase in ipsilateral forelimb use corresponding to the lesioned hemisphere may be expected in an established rat PD model, while a decrease in the percentage of ipsilateral use and a more symmetric forelimb use pattern are anticipated following effective treatment. Regarding the cylinder test, two-way ANOVA showed a significant main effect of group, F (5, 5) = 159.6, *p* < 0.001. In the analysis performed after dapagliflozin treatment, a trend toward decreased ipsilateral forelimb use was observed in all treatment groups compared with the PC group, the most pronounced reduction being detected in the G4 group (Fig. 3b).

The apomorphine-induced rotation test was performed 14 days after 6-OHDA administration in order to confirm establishment of the rat PD model. 6-OHDA administered into the right SNpc disrupts the nigrostriatal dopaminergic balance, leading to repetitive rotational behavior in the direction opposite (contralateral) to the lesioned hemisphere following apomorphine injection. The number of contralateral rotations is expected to decrease and approach normal levels following effective treatment. In post-model measurements, the number of rotations exceeded 100 in all groups, indicating marked motor asymmetry (Fig. 3c). Two-way ANOVA revealed a significant interaction between group and time, F (4, 50) = 28.09, *p* < 0.0001. Post hoc multiple-comparison analysis showed a significant increase in contralateral rotations in the PC group after model induction compared with the pre-treatment measurement (adjusted *p* < 0.0001). Among the dapagliflozin-treated groups, only G4 showed a significant reduction in contralateral rotations after treatment compared with its pre-treatment value (mean difference = 48.01, 95% CI 22.05 to 73.97, adjusted *p* < 0.0001). (Fig. 3c).

### Dapagliflozin attenuates 6-OHDA-induced pyknotic-degenerative neuronal changes

**Fig. 4:**
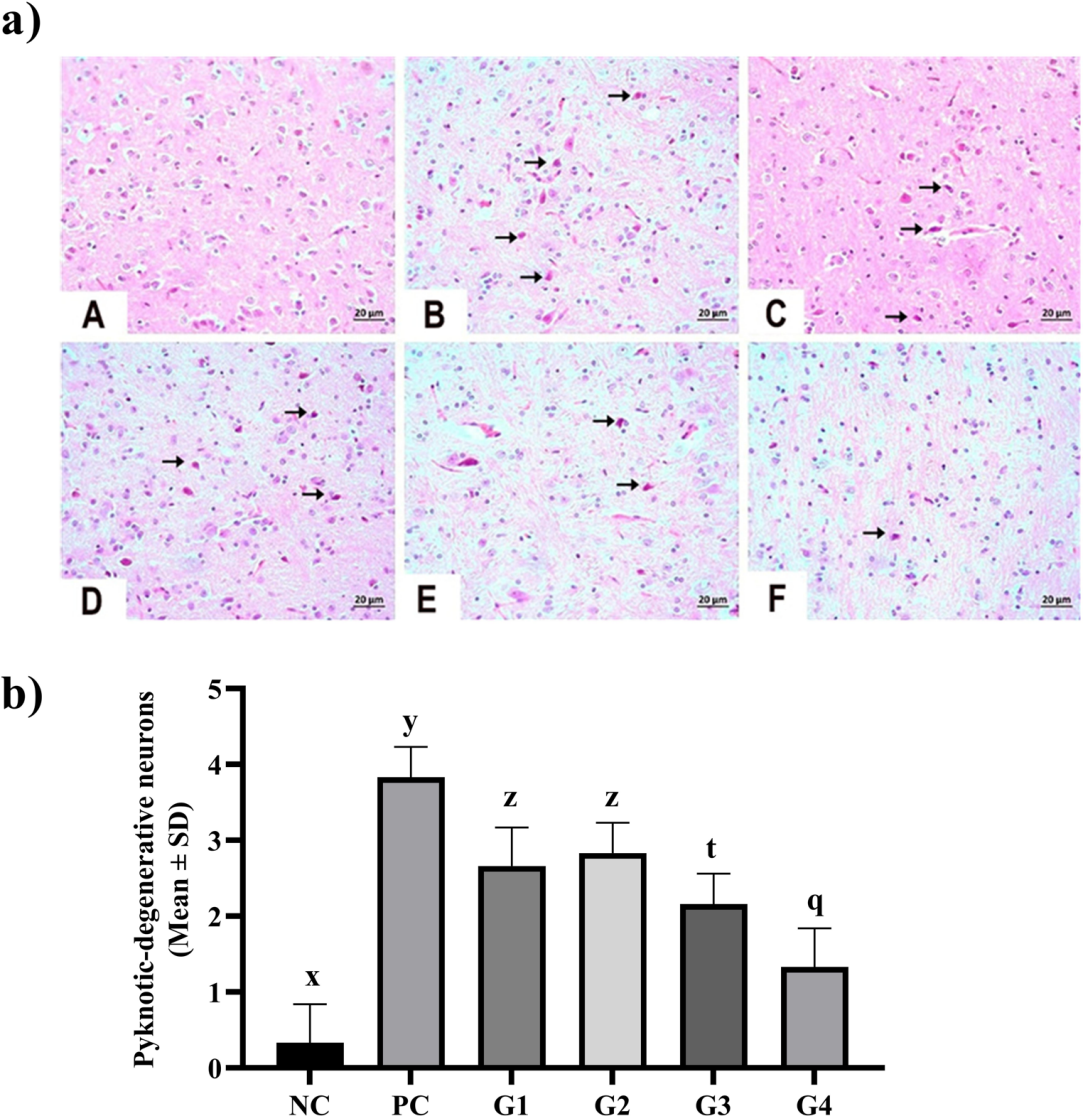
**a** Histopathological appearance of pyknotic-degenerative changes in brain tissue. (A) NC group; normal histological appearance. (B) PC group; very severe, (C) G1 group; severe, (D) G2 group; severe, (E) G3 group; moderate, (F) G4 group; mild. Pyknotic-degenerative neurons (→), H-E, Scale bar: 50 μm** b** Analysis of pyknotic-degenerative changes observed in neurons. Pyknotic-degenerative changes expressed as x: none, y: very severe, z: severe, t: moderate, q: mild indicate the difference between groups (*p* < 0.05). All data are presented as mean ± SD. Negative control (NC), positive control (PC), 6-OHDA + dapagliflozin 2.5 mg/kg (G1), 6-OHDA + dapagliflozin 5 mg/kg (G2), 6-OHDA + dapagliflozin 7.5 mg/kg (G3) and 6-OHDA + dapagliflozin 10 mg/kg (G4). (*p* < 0.05, Kruskal-Wallis test followed by Mann–Whitney U test)

Histopathological analysis revealed a normal histological appearance in the NC group, whereas neurons in the other groups exhibited pyknotic-degenerative changes of varying severity. Very severe pyknotic-degenerative changes were observed in the PC group, while these microscopic findings were classified as severe in the G1 and G2 groups, moderate in the G3 group, and mild in the G4 group. The nucleus and cytoplasm could not be clearly distinguished in neurons exhibiting pyknotic-degenerative changes, and the cells appeared dark and shrunken (Fig. 4).

### Dapagliflozin reduces α-synuclein and caspase-3 immunoreactivity and preserves TH immunoreactivity

**Fig. 5:**
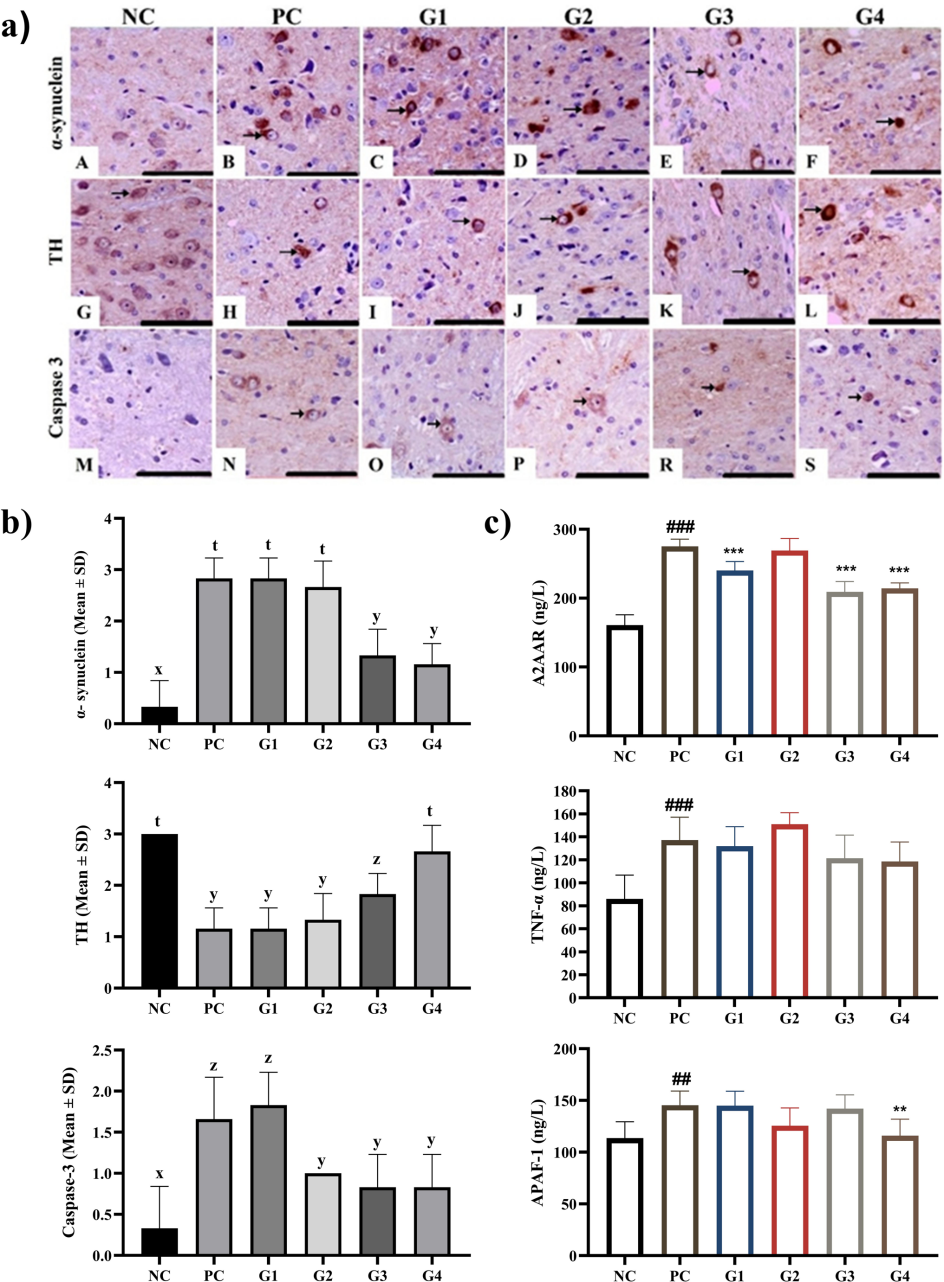
Effects of dapagliflozin treatment on α-synuclein, A2AAR (synonym ADORA2A), TH, TNF-α, APAF-1 and caspase-3 in the 6-OHDA-lesioned rat brain. **a** Immunohistochemical staining for α-synuclein (A–F), TH (G–L) and caspase-3 (M–S) in the brain. From left to right: NC, PC, G1, G2, G3 and G4 groups. The NC group shows almost no α-synuclein or caspase-3 immunoreactivity and strong TH staining, whereas the PC group shows intense α-synuclein accumulation, reduced TH expression and increased caspase-3 positivity. Dapagliflozin-treated groups (G1–G4) display decreased α-synuclein and caspase-3 staining and preservation of TH expression, particularly in G3 and G4. Arrows indicate immunopositive neurons (→). IHC, immunohistochemistry; scale bar = 50 μm. (*p* < 0.05, Kruskal-Wallis test followed by Mann–Whitney U test). **b** Statistical analysis of α-synuclein, TH and caspase-3 immunopositivity in brain tissue. Changes expressed as x: none, y: mild, z: moderate, t: severe indicate the difference between groups (*p* < 0.05). **c** A2AAR (synonym ADORA2A), TNF-α and APAF-1 levels in brain tissue. All data are presented as mean ± SD (*n* = 6 animals per group). ^##^*p* ≤ 0.01 and ^###^*p* ≤ 0.001 versus NC; ^*^p < 0.05, ^**^p ≤ 0.01, and ^*^p ≤ 0.001 versus PC. Negative control (NC), positive control (PC), 6-OHDA + dapagliflozin 2.5 mg/kg (G1), 6-OHDA + dapagliflozin 5 mg/kg (G2), 6-OHDA + dapagliflozin 7.5 mg/kg (G3) and 6-OHDA + dapagliflozin 10 mg/kg (G4) (One-way ANOVA with post-hoc Tukey test)

Immunohistochemical staining of rat brain tissues for α-synuclein, TH, and caspase-3 revealed statistically significant differences among the groups. In the NC group, no significant α-synuclein immunopositivity was detected, whereas severe α-synuclein immunopositivity was observed in the PC, G1, and G2 groups and mild immunopositivity in the G3 and G4 groups. TH immunopositivity was severe in the NC and G4 groups, moderate in the G3 group, and mild in the PC, G1, and G2 groups. In terms of caspase-3, no meaningful immunopositivity was observed in the NC group, while immunopositivity was moderate in the PC and G1 groups and mild in the G2, G3, and G4 groups (*p* < 0.05) (Fig. 5a, b).

### Dapagliflozin treatment reduces 6-OHDA-induced increases in A2AAR, TNF-α, and APAF-1 levels

One-way ANOVA analysis of A2AAR showed a significant difference among the groups, F (5, 30) = 60.30, *p* = 9.65 × 10^−15^. Bartlett’s test indicated homogeneity of variances (Bartlett’s statistic = 3.271, *p* = 0.66). Post hoc Tukey’s test showed that A2AAR levels were significantly higher in the PC group than in the NC group (mean difference = 114.3, 95% CI 90.67 to 138.0, adjusted *p* < 0.001). Compared with the PC group, A2AAR levels were significantly lower in G1 (mean difference = 35.00, 95% CI 11.34 to 58.66, adjusted *p* = 0.001), G3 (mean difference = 66.00, 95% CI 42.34 to 89.66, adjusted *p* < 0.001), and G4 (mean difference = 61.00, 95% CI 37.34 to 84.66, adjusted *p* < 0.001), whereas no significant difference was observed between the PC and G2 groups (mean difference = 6.000, 95% CI -17.66 to 29.66, adjusted *p* = 0.97) (Fig. 5c).

Analysis of TNF-α levels revealed a significant difference among the groups, as determined by one-way ANOVA [F (5, 30) = 9.215, *p* = 0.000021]. Bartlett’s test indicated homogeneity of variances (Bartlett’s statistic = 2.654, *p* = 0.75). TNF-α levels were significantly higher in the PC group than in the NC group (mean difference = 51.11, 95% CI 19.82 to 82.40, adjusted *p* = 0.00034). However, no significant differences were observed between the PC group and the dapagliflozin-treated groups, namely G1 (adjusted *p* > 0.99), G2 (adjusted *p* = 0.76), G3 (adjusted *p* = 0.65), and G4 (adjusted *p* = 0.48) (Fig. 5c).

For APAF-1, one-way ANOVA showed a significant difference among the groups, F (5, 30) = 5.743, *p* = 0.00078. Bartlett’s test indicated homogeneity of variances (Bartlett’s statistic = 0.5033, *p* > 0.99). Post hoc Tukey’s test showed that APAF-1 levels were significantly higher in the PC group than in the NC group (mean difference = 31.93, 95% CI 5.432 to 58.43, adjusted *p* = 0.01). Compared with the PC group, APAF-1 levels were significantly lower only in G4 (mean difference = 29.63, 95% CI 3.132 to 56.13, adjusted *p* = 0.02), whereas no significant differences were observed between the PC group and G1 (adjusted *p* > 0.99), G2 (adjusted *p* = 0.23), or G3 (adjusted *p* > 0.99). (Fig. 5c).

## Discussion

Dapagliflozin is a clinically well-known drug with several important advantages for repurposing in the treatment of PD. Clinical studies have reported that the antihyperglycemic dose of dapagliflozin ranges from 5 to 10 mg and that it is safe and well-tolerated up to 100 mg (Komoroski et al. 2009; Yang et al. 2013). In addition, owing to its low molecular weight and lipophilic nature, dapagliflozin has been observed to cross the BBB, a critical target for the effective treatment of neurodegenerative diseases (Nguyen et al. 2020; Rizzo et al. 2022). Current clinical and experimental studies provide notable evidence that SGLTis may alleviate neurodegeneration. However, studies investigating the effects of dapagliflozin on PD remain quite limited (Nguyen et al. 2020; Rizzo et al. 2022; NCT06263673, Mohammed et al. 2024). The present study investigated the neuroprotective effects of the SGLT2i dapagliflozin in an experimentally induced rat PD model. The results showed that daily treatment with 10 mg/kg dapagliflozin led to improvement in motor function in PD-modeled rats after 14 days and that the treatment produced clear and significant findings in terms of neuroprotection by reducing neuroinflammation.

The modeling of symptoms associated with human diseases in animals using various methods represents a practical and reliable approach for evaluating candidate drugs (Khan et al. 2024b). 6-OHDA is a neurotoxin that mimics the core pathological features of PD in humans through nigrostriatal dopaminergic degeneration and is used to evaluate the effects of candidate drugs on motor abilities (Altunlu et al. 2024). In the present study, pre-treatment measurements performed following model establishment revealed decreased vertical activity, ambulatory activity, and total distance traveled and, in parallel, a significant increase in resting time in the 6-OHDA-administered groups, compared with the NC group. This finding is consistent with other reports from the literature confirming that 6-OHDA induction causes a marked impairment in motor performance (Slézia et al. 2023; Aydın, 2026). The increased immobility and decreased locomotor activity induced by 6-OHDA were partially improved in the G4 (10 mg/kg) dapagliflozin treatment group in particular, and the drug exerted a dose-dependent restorative effect on motor dysfunction. The apomorphine-induced rotation test yields highly satisfactory results in determining striatal damage that leads to motor dysfunction. 6-OHDA induction disrupts the dopamine release process in the nigrostriatal system. Conversely, a slight increase is observed in the number of dopamine receptors in the striatum. Apomorphine, a dopamine agonist, binds to dopamine receptors in the striatum and elicits a contralateral turning response (Feng et al. 2021; Asemi-Rad et al. 2022; Forouzanfar et al. 2025). In our findings, the fact that all rats with 6-OHDA lesions exhibited more than 100 rotations following apomorphine administration indicates that PD-characterized dopaminergic loss was successfully established. Moreover, the reduction in rotation numbers in the G4 rats after dapagliflozin treatment suggests attenuation of striatal damage.

In the cylinder test used to assess the 6-OHDA lesion, the increase in right paw use decreased after dapagliflozin treatment. This finding was associated with attenuation of lesion-side motor asymmetry and partial restoration of dopaminergic system function. Compared with the PC group, a decreasing trend in terms of ipsilateral paw use was observed across all the treatment groups. However, since the most pronounced reduction occurred in the high-dose G4 (10 mg/kg) group, it may be assumed that relatively higher doses of dapagliflozin are required to elicit observable effects in the brain. Indeed, a study modeling experimental autism spectrum disorder in rats reported that 10 mg/kg dapagliflozin treatment for 15 days improved motor behaviors in the open field test, sociability test, and passive avoidance task compared with an untreated group (Erdogan et al. 2024). In a study involving an aluminum chloride–induced AD model, dapagliflozin treatment (1 mg/kg and 5 mg/kg) for 28 days was reported to significantly improve escape latency in the Y-maze and Morris water maze tests in the high-dose group and to yield higher scores for learning and memory (Samman et al. 2023). Considering that the effective dose range varies depending on model type, lesion severity, and duration of administration, the neuroprotective effective doses of dapagliflozin need to be systematically evaluated in clinical studies.

Elucidating the mechanisms through which repurposed drugs exert neuroprotective effects is of critical importance in terms of permitting earlier integration into clinical treatment and optimizing therapeutic regimens (Pekdemir et al. 2024). *In silico* analyses were therefore used to identify PD-associated target parameters that may be affected by dapagliflozin treatment in this study. Our STRING analyses revealed a significant interaction network among PD-related proteins, including α-synuclein, A2AAR, TH, TNF-α, APAF-1, and caspase-3. Targeting these proteins may be expected to yield an integrated neuroprotective response through the regulation of neuroinflammation, dopaminergic dysfunction, and apoptotic processes, which play critical roles in the pathophysiology of PD. Indeed, the molecular docking interaction scores of dapagliflozin with some of the identified target proteins were comparable to those of known inhibitors.

α-synuclein pathology is considered the gold standard for the diagnosis of PD and constitutes a critical therapeutic target. α-synuclein aggregation leads to neuroinflammation and mitochondrial dysfunction and is responsible for degeneration of dopaminergic neurons (Calabresi et al. 2023; Negi et al. 2024). Our molecular docking analysis showed that dapagliflozin exhibited a higher binding affinity to α-synuclein (−4.126 kcal/mol) than its known inhibitor (Emrusolmin, −3.726 kcal/mol). Immunohistochemical analyses revealed significantly lower α-synuclein immunopositivity in the SN region in rats receiving 7.5 mg/kg and 10 mg/kg dapagliflozin compared with the PC group. These results also support our findings of improved motor function. Due to its relationship with α-synuclein, its effects on the dopamine D2 receptor, and the fact that increased expression triggers neurodegeneration, A2AAR has been proposed as a potential therapeutic target in PD. A study involving monozygotic twins sharing the same genetic background and living under similar environmental conditions reported that A2AAR expression was 1.8-fold higher in twins with PD than in healthy twins (Semenova et al. 2022). In addition to the reported sporadic cases, experimental models have also demonstrated that α-synuclein aggregation is associated with increased A2AAR expression. Intracerebral induction of mutant α-synuclein fibrils has been shown to increase A2AAR expression in the mouse hippocampus (Hu et al. 2016). Increased A2AAR expression has also been reported in previous studies using a 6-OHDA-induced experimental PD model (Falconi et al. 2019). Our biochemical analysis revealed decreased A2AAR levels in brain tissue in all the treatment groups compared with the PC group. Although a high dapagliflozin-A2AAR binding affinity was revealed by *in silico* analyses (−7.387 kcal/mol), no significant difference was identified among the treatment doses.

Regulation of dopaminergic pathways, including those involving D2 and A2AAR, improves involuntary movements and motor control (Mori et al. 2022; Pinna et al. 2018). Levels of TH, the rate-limiting step in dopamine synthesis, are lower in individuals with PD, and this represents one of the early features associated with the pathogenesis of the disease (DiFrancisco-Donoghue et al. 2014). With increasing progression and neurodegeneration, the decline in TH activity further exacerbates α-synuclein pathology. Studies of induced PD models in animals and in human brain tissues have reported a significant negative correlation between TH levels and disease stage (Chotibut et al. 2014; Rausch et al. 2022). In the present study, TH loss in the SN region was lower in rats treated with 10 mg/kg dapagliflozin. In addition, the binding score of dapagliflozin to TH (−5.476 kcal/mol) was very close to that of a known TH inhibitor (metyrosine, −5.605 kcal/mol).

TNF-α, which plays an important role in neuroinflammation, is a proinflammatory cytokine secreted by microglial cells. Microglial activation and increased TNF-α levels in the SNpc trigger an inflammatory response in experimental PD models, particularly following 6-OHDA or rotenone administration (Chertoff et al. 2011; Preeti et al. 2023). Studies have shown that dapagliflozin can regulate inflammation in brain tissue (Nguyen et al. 2020; Erdogan et al. 2024). Erdoğan et al. reported that TNF-α and IL-17 levels, which increased following induction of autism spectrum disorder, decreased after dapagliflozin treatment (Erdogan et al. 2024). In our molecular docking analysis, the binding score between TNF-α and dapagliflozin was − 5.771 kcal/mol, while biochemical analyses revealed significantly decreased TNF-α levels in rat brain tissues at doses of 7.5 mg/kg and 10 mg/kg, compared with the PC group. These data provide promising evidence that dapagliflozin may interact via TNF-α receptors. Although it has not yet been sufficiently investigated in patients with PD, APAF1 is considered a novel therapeutic target for neurodegenerative diseases due to its role in triggering the apoptotic process that develops with oxidative stress and in initiating the caspase cascade (Luchetti et al. 2023; Li et al. 2025). A study evaluating the expression levels of 52 genes associated with oxidative stress and inflammation in peripheral blood mononuclear cells from 48 patients with PD and 25 healthy controls reported higher APAF-1 levels compared with healthy individuals. Moreover, elevation in APAF-1 levels was correlated with scores registered on scales used to determine PD symptoms and disease progression (Chang et al. 2023). APAF-1 expression and levels of oxidative stress-related parameters in an experimental PD model induced by subcutaneous MPTP injection in mice in another study were significantly lower in pramipexole-treated groups compared with the PC group (Wang et al. 2018). In the present study, APAF-1 levels decreased markedly in the groups treated with 5 mg/kg and 10 mg/kg dapagliflozin. Additionally, the dapagliflozin-APAF-1 binding affinity in molecular docking analyses (−5.802 kcal/mol) was associated with the potential of dapagliflozin to modulate apoptotic pathways in brain tissue.

Caspase-3, a key component of the caspase cascade, is one of the principal mediators of neuronal apoptosis. Experimental studies have reported that caspase-3 activation is required for the development of PD. Moreover, postmortem analyses of brains from patients with PD have shown an increased number of neurons expressing active caspase-3 in the SNpc region (Hartmann et al. 2000; Yamada et al. 2010; García-Revilla et al. 2024; Choi et al. 2024). A study involving a rotenone-induced PD model in rats showed that dapagliflozin treatment reduced apoptosis in dopaminergic neurons. This effect was associated with decreased expression of the pro-apoptotic Bax and active caspase-3 proteins (Arab et al. 2021). Dapagliflozin treatment was also reported to reduce caspase-3 expression in brain tissue and suppress apoptosis in a lipopolysaccharide-induced (LPS) AD model (Abd Elmaaboud et al. 2023). In the present study, caspase-3 immunopositivity decreased in the G2, G3, and G4 groups compared with the PC group. In this process, dapagliflozin may have alleviated neurodegeneration by suppressing apoptotic activation. In the light of this and other studies in the literature, dapagliflozin appears to affect apoptosis across different disease models by modulating caspase-3 (Arab et al. 2021; Abd Elmaaboud et al. 2023; Dos Santos et al. 2025). In addition, these effects seem to vary depending on the disease type and the cellular signaling pathways targeted by dapagliflozin.

### Limitations

This study has some limitations that should be acknowledged. Assessing the drug effects in only one sex (male or female) prevents evaluation of potential sex-dependent differences in the neuroprotective effects of treatment; therefore, the findings should be interpreted with caution in terms of generalizability. Another limitation of the present study is that, although the potential effects of dapagliflozin in the experimental PD model were comprehensively evaluated using *in silico*, histopathological, immunohistochemical, and biochemical approaches, the obtained findings were not corroborated by molecular techniques such as Western blotting and quantitative PCR. In addition, dapagliflozin was administered for 14 days, so the study reflects only short-term effects. Therefore, there is a need for longer-term experimental and clinical studies to determine the long-term effects of dapagliflozin in PD.

## Conclusion

The present study demonstrated that dapagliflozin may exert potential neuroprotective effects in an experimental PD model by modulating pathways associated with neuroinflammation, oxidative stress, and apoptosis. In particular, dapagliflozin reduced the expression of α-synuclein, A2AAR, TNF-α, APAF-1, and caspase-3, while modulating motor impairment through regulation of dopaminergic pathways. These findings suggest that dapagliflozin may represent a promising repurposing candidate for PD management. However, further preclinical and clinical studies are needed to confirm these effects and clarify the underlying mechanisms.

## Data Availability

Data will be made available on request.

## Abbreviations

6-OHDA: 6-hydroxydopamine
A2AAR: Adenosine A2A receptor
AD: Alzheimer’s disease
AP: Anteroposterior
APAF-1: Apoptotic protease-activating factor-1
BBB: Blood-brain barrier
COMT: Catechol-O-methyltransferase
DAB: 3,3′-Diaminobenzidine
DV: Dorsoventral
FDR: False discovery rate
GO: Gene ontology
IHC: Immunohistochemistry
L: Lateral
L-DOPA: L-3,4-dihydroxyphenylalanine
LPS: Lipopolysaccharide
MAO: Monoamine oxidase
MPTP: 1-methyl-4-phenyl-1,2,3,6-tetrahydropyridine
PBS: Phosphate-buffered saline
PD: Parkinson’s disease
PPI: Protein-protein interaction
SD: Standard deviation
SN: Substantia nigra
SNpc: Substantia nigra pars compacta
SGLT: Sodium-glucose cotransporter
SGLT2is: Sodium-glucose cotransporter 2 inhibitor
TH: Tyrosine hydroxylase
TNF-α: Tumor necrosis factor-alpha
WHO: World Health Organization
